# Mutation in *Wzz(fepE)* Linked to Altered O-Antigen Biosynthesis and Attenuated Virulence in Rough *Salmonella* Infantis Variant

**DOI:** 10.3390/vetsci11120603

**Published:** 2024-11-28

**Authors:** Nneka Vivian Iduu, Steven Kitchens, Stuart B. Price, Chengming Wang

**Affiliations:** Department of Pathobiology, College of Veterinary Medicine, Auburn University, 1130 Wire Road, Auburn, AL 36849-5519, USA; nvi0001@auburn.edu (N.V.I.); srk0002@auburn.edu (S.K.); pricesb@auburn.edu (S.B.P.)

**Keywords:** *Salmonella* Infantis, virulence, O-antigen chain regulator, *Wzz(fepE)*, rough variant

## Abstract

*Salmonella enterica* serovar Infantis has emerged as a prevalent foodborne pathogen in poultry with significant global health implications. This study investigates the molecular characteristics influencing virulence in a *S.* Infantis rough variant collected from a poultry farm in the USA. In this study, whole genome sequencing and comparative genomics were performed on smooth and rough poultry *S.* Infantis isolates, while chicken embryo lethality assay was conducted to assess their virulence. Comparative genomics between isolates was analyzed using Mauve pairwise Locally Collinear Blocks to measure the genetic conservation. Embryo survival rates between the isolates were compared using the Kaplan–Meier curves. High genomic conservation was observed between the two isolates, but a frameshift mutation was detected in the *Wzz(fepE)* gene of the rough variant, resulting in early protein truncation. The chicken embryo lethality assay showed that the lethality rate of the smooth strain was higher than that of the rough strain (*p* < 0.05). This study identifies a frameshift mutation in the *Wzz(fepE)* gene, leading to protein truncation, which may reduce bacterial virulence by impacting O-antigen biosynthesis in the rough *Salmonella* Infantis variant. These findings deepen our understanding of *S.* Infantis pathogenesis and suggest that targeting the *Wzz(fepE)* gene or related pathways could be a promising strategy for developing effective vaccines and therapeutic interventions.

## Simple Summary

This study investigates a rough variant of *Salmonella* Infantis, a common foodborne pathogen in poultry that can cause illness in humans. Researchers compared a smooth and rough version of the bacteria collected from poultry farms in the USA. Using genetic analysis, they found that both versions were highly similar, but the rough variant had a mutation in a gene called *Wzz(fepE)*. This mutation affected the bacteria’s ability to produce O-antigen, a structure important for virulence. We tested how harmful each version was using a chicken embryo model. The smooth strain was more lethal to embryos, while the rough strain showed reduced virulence. This suggests that the mutation in the rough strain may weaken its ability to cause disease. These findings enhance our understanding of *Salmonella* Infantis and could help in developing new vaccines or treatments to control infections.

## 1. Introduction

*Salmonella enterica* subsp. *enterica* serovar Infantis (antigenic formula 6,7,14:r:1,5) has emerged as a prevalent serovar in poultry, contributing to human salmonellosis [1]. *Salmonella* Infantis typically causes non-life-threatening episodes of gastroenteritis. However, certain strains may exhibit enhanced virulence, enabling them to cause invasive infections that can progress beyond gastroenteritis [2]. Recent years have seen a concerning rise in its prevalence, marked by increased virulence, conferring enhanced environmental fitness [2,3].

Understanding the virulence factors of *S.* Infantis is essential for controlling its transmission and impact on human health. Like other *Salmonella* serovars, the persistence and virulence of *S.* Infantis are significantly influenced by its surface structures, particularly lipopolysaccharide (LPS), which accounts for about 70% of its outer membrane [4,5]. LPS consists of three components: Lipid A, a core oligosaccharide, and the O-antigen. The O-antigen, a polysaccharide of variable chain lengths and composition, forms the outermost part of LPS and plays a crucial role in host–pathogen interaction and immune evasion [4].

The genes involved in O-antigen biosynthesis include those responsible for synthesizing nucleotide sugar precursors specific to the O-antigen, sugar transferase genes for O-unit assembly, and O-unit processing genes that regulate translocation and chain polymerization [6]. These genes are typically organized in a gene cluster located between *galF* and *gnd* in *Salmonella*, although some genes responsible for structural modifications, such as the adding side-chain residues, may be located outside this cluster. [6]. In *Salmonella* Enteritidis and *Salmonella* Typhimurium, the O-antigen chain length follows a modal distribution, regulated by the *Wzz* genes: *WzzST* (also known as *WzzB*) regulates the synthesis of long O-antigen chains of 16–35 repeat units, while *Wzz(fepE)* regulates the synthesis of very long O-antigen chain >100 repeat units [4]. Studies indicate that the distribution of O-antigen chain lengths on the outer membrane enables *Salmonella* to escape host immune responses, avoid complement-mediated killing, and promote bacterial persistence in the host [4]. Consequently, O-antigen chain length regulators play an important role in *S.* Typhimurium virulence, highlighting the O-antigen as a critical determinant of pathogenesis [7,8,9].

However, the specific genetic determinants governing O-antigen biosynthesis and their functional impact on *S.* Infantis virulence remain largely unexplored. O-antigen diversity is essential for bacterial serotyping, which helps in identifying *Salmonella* strains that vary in host range and disease spectrum [8]. *Salmonella* serotyping is based on the antigenic formula, which includes both the O-antigen and flagellar (H) antigens [8]. Recent studies have highlighted the emergence of rough variants of *S.* Infantis with incomplete antigenic formulas. These variants carried flagellar antigens typical of *S.* Infantis (-:r:1,5) but lacked the O-antigen (6,7) [10,11].

Here, we characterize the genomic and phenotypic attributes of a rough *S.* Infantis variant to decipher the genetic alterations influencing its virulence by utilizing sequencing, bioinformatics analysis, and chicken embryo lethality assay. This study advances our understanding of *S.* Infantis pathogenicity and opens new avenues for targeted interventions and vaccine development against this emerging pathogen.

## 2. Materials and Methods

### 2.1. Bacterial Strains

Two *Salmonella enterica* isolates were used in this work. One isolate, designated as *Sal*_smooth, was a *Salmonella enterica* serovar Infantis strain from a poultry production facility in the state of Alabama. Another isolate, designated as *Sal*_rough, was a *Salmonella* Infantis -:r:1,5 from a boot swab at a pullet farm located in the state of Alabama.

### 2.2. Whole Genome Sequencing (WGS) and Bioinformatics Analysis

Genomic DNA of *Sal*_rough and *Sal*_smooth was extracted from pellet obtained from 1.0 mL of overnight culture grown in Luria–Bertani (LB) broth using the Dneasy Blood and Tissue Kit (Qiagen, Germantown, MD, USA) following the manufacturer’s protocol. Long-read whole genome sequencing was performed by Plasmidsaurus (Eugene, OR, USA) using Oxford Nanopore Technologies (ONT) PromethION. ONT long reads were quality-filtered using Filtlong v0.2.1 (https://github.com/rrwick/Filtlong, accessed date: 10 September 2024), and de novo assembly was performed using Flye v2.9.4 (https://github.com/mikolmogorov/Flye, [12], accessed date: 10 September 2024). The assemblies were further polished with Illumina reads (generated on Illumina NextSeq2000) using Polypolish v0.6.0 (https://github.com/rrwick/Polypolish/releases, accessed date: 10 September 2024) to generate hybrid assemblies. Genome completeness and contamination were assessed using CheckM v1.2.2 (https://github.com/Ecogenomics/CheckM [13], accessed date: 10 September 2024). The genome annotations were performed with NCBI Prokaryotic Genome Annotation Pipeline (PGAP) (https://github.com/ncbi/pgap, accessed date: 10 September 2024).

Serotyping of both *S*. Infantis isolates was confirmed using in silico serotyping methods on the NCBI pathogen detection webpage (https://www.ncbi.nlm.nih.gov/pathogens/isolates, accessed date: 10 September 2024). Sequence type was determined with in silico multi-locus sequence typing analysis (MLST) (https://github.com/tseemann/mlst, [14]). Multiple genome alignment was carried out with Mauve software (https://darlinglab.org/mauve/mauve.html, accessed date: 10 September 2024). Plasmids were identified using the PlasmidFinder database on ABRicate (https://github.com/tseemann/abricate [15], accessed date: 10 September 2024).

### 2.3. Identification of O-Antigen Biosynthesis Genes and Wzz(fepE) Mutation in S. Infantis

The assembled genomes of *Sal*_smooth and *Sal*_rough were compared to identify genetic variants potentially responsible for possible phenotypic differences between the strains. Specific attention was given to genes involved in O-antigen biosynthesis and regulation, as these are known to influence virulence. The genes involved in O-antigen biosynthesis for *S.* Infantis was identified by performing a nucleotide BLAST between the Seroseq serotype determinant database (rfb cluster database) from SeqSero2 (http://www.denglab.info/SeqSero2 [16], accessed date: 10 September 2024) and *S.* Infantis reference genomes on NCBI database. Additional O-antigen biosynthesis genes were identified through annotation. The identified genes were compared with those found in *Sal*_smooth to *Sal*_rough genomes. *Wzz(fepE*) mutation was detected by reference mapping *Sal*_smooth to *Sal*_rough genome with BWA/MEM v2.2.1 (https://github.com/bwa-mem2/bwa-mem2 [17], accessed date: 10 September 2024), variant calling was performed using snippy v4.6.0 (https://github.com/tseemann/snippy [18], accessed date: 10 September 2024), and variant visualization was conducted with Integrated Genome Viewer (IGV) v2.17.3 (https://igv.org/doc/desktop/#DownloadPage/ [19], accessed date: 10 September 2024). Confirmation of mutation was achieved through NCBI BLAST (http://www.ncbi.nlm.nih.gov/BLAST, accessed date: 10 September 2024) and additional annotation.

### 2.4. PCR and Sanger Sequencing

To further confirm the frameshift mutation in the *Wzz(fepE)* gene of *Sal*_rough, Sanger sequencing was performed. Genomic DNA was extracted from the culture of *Sal*_rough, its 20th days of serial passages variant, and *Sal*_smooth strain using Dneasy Blood and Tissue Kit (Qiagen, Germantown, MD, USA) following the manufacturer’s instructions. PCR targeting the mutation in the O-antigen length regulator *(Wzz(fepE)**)*** gene of the *S.* Infantis rough strain was conducted on a Roche light cycler 480 II system (Roche Molecular Biochemicals, Indianapolis, IN, USA). The primers (synthesized by Integrated DNA Technologies, Coralville, IA, USA) are as follows: forward primer: 5′-AAA CAG ATT AAA TAC GCT GGC CCG A 3′; reverse primer: 5′-GGC GCG TAA AGA TTG TTT CGG ATA A-3′. The products of *Wzz(fepE)* PCR were sent to ELIM Biopharmaceuticals (Hayward, CA, USA) for Bidirectional Sanger sequencing. Chromatograms were analyzed and visualized using Unipro UGENE v50.0 (https://ugene.net/, accessed date: 10 September 2024).

### 2.5. Chicken Embryo Lethality Assay

The virulence of *S.* Infantis isolates was assessed using a modified chicken embryo lethality assay [20]. SPF-embryonated eggs (AVSBio, Norwich, CT, USA) were incubated at 37 °C with 65% humidity until day 11. On day 12, fertile eggs were marked and divided into three groups: two experimental (12 eggs each) and one negative control (10 eggs) groups. Experimental groups included *Sal*_smooth, *Sal*_rough, and its 20th days of serial passages variant. The *S*. Infantis isolates were cultured overnight in LB broth, and the inoculum concentration was adjusted to 10^3^ CFU/mL. After surface sterilization with 70% ethanol, eggs were inoculated with 100 µL of bacterial suspension or sterile Dulbecco’s phosphate-buffered saline (PBS) as negative control into the allantoic cavity. Inoculation sites were sealed with paraffin wax. Eggs were incubated at 37 °C and candled daily to monitor embryo mortality until day 16 of development (5 days post-inoculation). Embryo death occurring within 24 h of inoculation was considered non-specific and attributed to lethal trauma caused by the inoculation. The experiment was carried out in two biological replicates to ensure the reproducibility of the results.

### 2.6. Statistical Analysis

Comparative genomics between isolates was analyzed using Mauve pairwise Locally Collinear Blocks (LCBs) to measure the genetic conservation. Embryo survival rates were compared between the isolates using Kaplan–Meier curves generated on GraphPad Prism v10.3.0 (461) (GraphPad Software Inc., Boston, MA, USA). Statistical analyses to compare groups were performed using SAS Studio v.3.81 (SAS Institute Inc., Cary, NC, USA), and differences were evaluated using the log-rank test. *p* values below 0.05 were considered statistically significant.

## 3. Results

### 3.1. Serotyping Confirms the Antigenic Formula of Sal_Smooth and Sal_Rough Strains

In silico serotyping confirmed the *Sal*_smooth strain as a typical antigenic formula, 7:r:1,5, and the *Sal*_rough maintained an antigenic formula of -:r:1,5. Both strains were identified as sequence type 32 (ST32), providing a foundation for comparative genomic analysis.

### 3.2. High Genomic Conservation Between Sal_Smooth and Sal_Rough Strains

**Figure 1:**
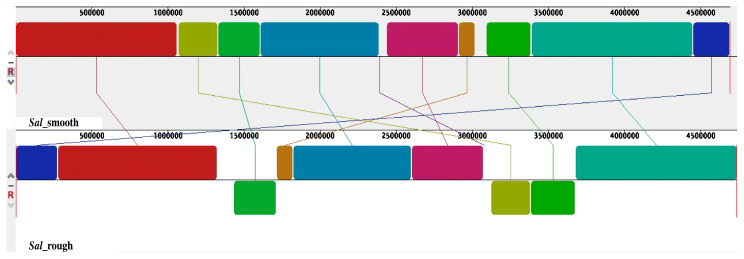
High genomic similarities between the whole genome sequences of *Sal*_smooth and *Sal*_rough. Pairwise alignment of the *Sal*_smooth and *Sal*_rough genomes was conducted using Mauve software. Colored blocks represent homologous (similar) regions, with connecting lines indicating shared sequences between the two genomes. Blocks below the center line indicate regions aligned in reverse complement (inverse) orientation. These homologous regions are called Locally Collinear Blocks (LCBs). A total of ten (*n* = 10) LCBs were identified, with a minimum weight of 3099, indicating strong homology and high similarity between the strains.

Whole genome comparison revealed a high level of conservation between *Sal*_smooth and *Sal*_rough, with 10 homologous Locally Collinear Blocks (LCBs) identified, with a minimum weight of 3099. The genomes of both strains were approximately 4.7 million base pairs in length (Figure 1). However, plasmid analysis revealed that *Sal*_rough variant harbored a 217,909 bp contig with the IncFIB(pN55391) plasmid replicon and yersiniabactin virulence factors encoded by the *fyuA*, *irp2-irp1-ybtUTE*, *ybtA*, and *ybtPQXS* gene cluster, while no plasmid was detected in *Sal*_smooth strain.

### 3.3. Frameshift Mutation in Wzz(fepE) Gene of Sal_Rough Strain

The analysis of the O-antigen biosynthesis genes in *S.* Infantis revealed that the region between the *galF* and *gndA* genes exhibited 99.6% sequence identity (9352 out of 9389 nucleotides) with the O-antigen gene cluster of *S.* Choleraesuis (serogroup C1, O-7 antigen) from the Seroseq serotype determinant database. This region in *S.* Infantis included several genes: *EpsG*, three glycosyltransferase genes, mannose-1-phosphate guanylyltransferase/mannose-6-phosphate isomerase *(manC)*, phosphomannomutase *(manB)*, and a hypothetical protein. Additional genes involved in O-antigen biosynthesis were identified outside this cluster, including UDP-N-acetylglucosamine–undecaprenyl-phosphate, N-acetylglucosamine phosphotransferase *(WecA)*, O-antigen ligase (*rfaL)*, and O-antigen length regulators (*WzzB*, *and Wzz(fepE)).* Comparative analysis between the *Sal*_rough and *Sal*_smooth genomes revealed the loss of several O-antigen biosynthesis genes: *EpsG* and the glycosyltransferases within the cluster in both genomes.

**Figure 2:**
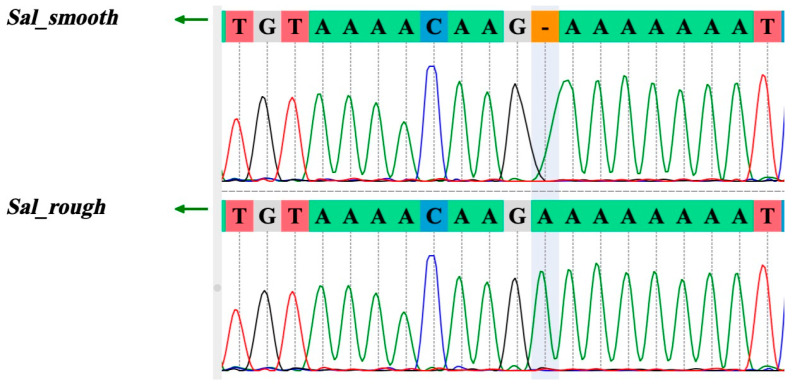
Frameshift mutation in the *Wzz(fepE)* gene of *S.* Infantis rough strain. The grey highlighted chromatogram region shows an adenine (A) insertion after nucleotide position 32, extending the gene length in *Sal*_rough from 1137 to 1138 bp as observed in *Sal*_smooth. This insertion introduces an early stop codon at amino acid position 26, truncating the protein from 378 to 25 amino acids.

The most notable difference was a frameshift mutation in the *Wzz(fepE)* gene of *Sal*_rough, caused by an adenine (A) insertion after nucleotide position 32, extending its length from 1137 bp to 1138 bp (Figure 2). This mutation resulted in an early stop codon at position 26 in the 378 amino acid *Wzz(fepE)* protein, causing truncation that renders it a pseudogene. Importantly, sequencing analysis further confirmed that the *Wzz(fepE)* mutation, characterized by the inserted adenine base, was present in *Sal*_rough but absent in *Sal*_smooth (Figure 2). Consequently, this mutation was unique to *Sal_rough* and remained stable over 20 days of serial passages.

### 3.4. Sal_Rough Demonstrates Attenuated Virulence in Chicken Embryo Model

**Figure 3:**
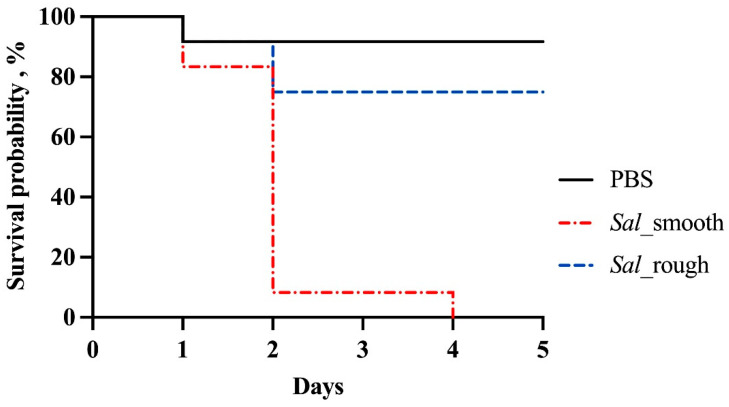
Chicken embryo lethality rate of *S.* Infantis strains. Twelve (12) eggs were used in each experimental group except the negative control (10 eggs used) with an inoculum concentration of 10^3^ CFU/mL and sterile Dulbecco’s phosphate-buffered saline (PBS) for the control group. Kaplan–Meier plot displaying the chicken embryo lethality when inoculated with sterile PBS (black line), smooth strain *Sal*_smooth (red line), and rough variant *Sal*_rough (blue line), for 5 days post-inoculation. Results show that the embryo lethality rate of the *Sal*_smooth group was higher than the negative control group and *Sal*_rough (*p <* 0.05). No difference in embryo lethality rates was observed between the negative control and *Sal*_rough (*p* = 0.721). Statistical analysis was performed using the log-rank test.

The chicken embryo lethality assay revealed significantly higher embryo lethality rates in the *Sal*_smooth group compared to both the negative control (PBS) and *Sal*_rough (*p* < 0.05). Notably, no significant difference in embryo lethality was observed between *Sal*_rough and the negative control (*p* = 0.721) (Figure 3), confirming the attenuated virulence of the rough variant. Additionally, no difference in virulence was observed between the original *Sal*_rough and its 20th days of serial passages variant (*p* = 0.2388), suggesting stable attenuation across repeated passages and multiple bacterial generations.

## 4. Discussion

*Salmonella enterica serovar* Infantis is an important zoonotic pathogen with rising prevalence and virulence [21,22,23]. While studies have investigated the role of O-antigen in *Salmonella* virulence [7,8,9], little is known about its regulatory mechanism in *S.* Infantis virulence. In this study, genomic and phenotypic comparison between rough (*Sal*_rough) and smooth (*Sal*_smooth) *S.* Infantis strains revealed that though both strains showed loss of the same O-antigen biosynthesis genes, the most significant finding was a frameshift mutation in the *Wzz(fepE)* gene of the rough variant, resulting in a truncated, non-functional protein that likely disrupted very long O-antigen chain synthesis. This finding aligns with previous studies by Tran and Morona et al. [24] on *FepE*, a *Wzz(fepE)* homolog in *Escherichia coli*, where mutations led to shorter O-antigen chain lengths. The presence of *Wzz(fepE)* homologs across *Salmonella* serovars and *E. coli* suggests a conserved role in the *Enterobacteriaceae* family [7]. This alteration likely explains the incomplete antigenic profile (-:r:1,5) in *Sal*_rough and influences its virulence due to impaired resistance to the immune response [25,26].

The chicken embryo lethality assay results provided evidence for the impact of a non-functional *Wzz(fepE)* gene in the virulence characteristics of the rough variant. The significantly lower embryo lethality rates observed in the *Sal*_rough group compared to *Sal*_smooth, and the lack of difference between *Sal*_rough and the negative control, confirm the reduced virulence of the rough variant. These findings are consistent with previous reports by Bravo et al. [27], who suggested that *Wzz(fepE)* in non-typhoidal *Salmonella* serovars such as *S.* Typhimurium primarily confers protection against serum bactericidal activity. These authors also demonstrated that since *S.* Typhi lacks a functional *Wzz(fepE)*, it was significantly less resistant to serum compared to *S.* Typhimurium. In *S.* Paratyphi, very long O-antigen chains also mediate immune evasion, though through a mechanism distinct from non-typhoidal *Salmonella* [28,29].

In an earlier study, Murray et al. [7] observed functional redundancy between O-antigen chain length regulators *Wzz(fepE)* and *WzzB* in *S.* Typhimurium C5 mutant, where either gene provided complement resistance and virulence in mice. However, although *Sal_rough* retained the *WzzB* gene, it showed reduced virulence, suggesting that functional *Wzz(fepE)* is critical for virulence in this strain. This aligns with another study by Murray et al. [8] showing that serum resistance in *S.* Typhimurium depends on *Wzz(fepE)*.

Consequently, studies by Pescaretti et al. [30] and da Silva et al. [31] highlighted the essential role of very long O-antigen chains in serum resistance and bacterial survival within macrophages during *Salmonella* pathogenesis. Crawford et al. [32] found that strains with functional *Wzz(fepE)* showed higher fitness in mouse colitis models, as evidenced by increased fecal bacterial counts. This advantage was attributed to enhanced bile salt resistance during inflammation, indicating that *Wzz(fepE)*-mediated very-long-O-antigen chain production contributes to *Salmonella* virulence.

The rough variant’s attenuation attributed to the *Wzz(fepE)* mutation makes it a promising candidate for live attenuated vaccine development, aligning with approaches proposed by Galen and Curtiss [33] for using attenuated strains in vaccine production. Modifying the O-antigen length has shown potential for enhancing immunogenicity and addressing T-independent immune responses [34,35]. Han et al. [36] have demonstrated that shortening O-antigen length impacts *Salmonella* immunogenicity. Thus, targeting *Wzz(fepE)* could lead to effective vaccines against *S.* Infantis.

Plasmid analysis revealed the presence of the IncFIB(pN55391) plasmid replicon and associated yersiniabactin virulence factors in the *Sal*_rough strain but not in *Sal*_smooth. The IncFIB(pN55391) plasmid replicon, typical of the PESI-like megaplasmid, has been identified in *S.* Infantis strains including ST32 [37,38], where these strains showed enhanced fitness and bacterial virulence, supporting *Salmonella* survival in animal hosts [2]. However, its presence in the *Sal*_rough strain did not exhibit any additional virulence effects, raising questions about the overall role of IncFIB(pN55391)-encoded factors in *Salmonella* virulence. Further research is needed to understand how IncFIB-encoded factors contribute to *Salmonella* pathogenesis and fitness across different strains [39].

The chicken embryo lethality assay serves as a well-established model for assessing the pathogenicity and virulence of avian pathogens [40,41]. While valuable for studying virulence, incorporating diverse animal models would provide a more comprehensive understanding of the strain’s attenuated characteristics.

One limitation of this study is that the causal relationship between the mutation in the *wzz(fepE)* gene and reduced pathogenicity requires further confirmation. Future work will include biochemical assays to quantify O-antigen levels in both rough and smooth *Salmonella* Infantis strains, imaging studies to observe potential physical differences in bacterial surface structures, and Western blotting to assess variations in immune recognition. Similar studies could be conducted on other Salmonella serotypes, such as *S*. Typhimurium and *S*. Enteritidis.

## 5. Conclusions

This study identifies a frameshift mutation in the *Wzz(fepE)* gene leading to early protein truncation, which may reduce bacterial virulence by impacting O-antigen biosynthesis. The reduced bacterial virulence, likely due to impaired O-antigen biosynthesis, suggests that targeting *Wzz(fepE)* could be a promising approach for developing attenuated *S.* Infantis vaccines. These findings expand our understanding of virulence mechanisms in *S.* Infantis and may inform strategies for controlling its infections. Future studies will employ CRISPR/Cas9 gene editing to validate the role of *Wzz(fepE)* in *S.* Infantis virulence.

## Data Availability

The nucleotide sequence data reported in this paper have been submitted to the NCBI Submission (National Library of Medicine, 8600 Rockville Pike, Bethesda, MD 20894) Nucleotide Sequence Database and have been assigned the accession numbers GCA_038020145 and GCA_038019735 under BioProject accession number PRJNA1090139.
